# Cyclin M2 (CNNM2) knockout mice show mild hypomagnesaemia and developmental defects

**DOI:** 10.1038/s41598-021-87548-6

**Published:** 2021-04-15

**Authors:** Gijs A. C. Franken, Murat Seker, Caro Bos, Laura A. H. Siemons, Bram C. J. van der Eerden, Annabel Christ, Joost G. J. Hoenderop, René J. M. Bindels, Dominik Müller, Tilman Breiderhoff, Jeroen H. F. de Baaij

**Affiliations:** 1 Department of Physiology, Radboud Institute for Molecular Life Sciences, Radboud University Medical Center, P.O. Box 9101, 6500HB Nijmegen, The Netherlands; 2 Department of Pediatric Gastroenterology, Nephrology and Metabolic Diseases, Charité – Universitätsmedizin Berlin, Berlin, Germany; 3 Department of Internal Medicine, Erasmus Medical Center, Rotterdam, The Netherlands; 4 Department of Molecular Cardiovascular Research, Max-Delbrueck-Center for Molecular Medicine, Berlin, Germany

**Keywords:** Kidney, Neurogenesis

## Abstract

Patients with mutations in Cyclin M2 (CNNM2) suffer from hypomagnesaemia, seizures, and intellectual disability. Although the molecular function of CNNM2 is under debate, the protein is considered essential for renal Mg^2+^ reabsorption. Here, we used a *Cnnm2* knock out mouse model, generated by CRISPR/Cas9 technology, to assess the role of CNNM2 in Mg^2+^ homeostasis. Breeding *Cnnm2*^+*/−*^ mice resulted in a Mendelian distribution at embryonic day 18. Nevertheless, only four *Cnnm2*^*−/−*^ pups were born alive. The *Cnnm2*^*−/−*^ pups had a significantly lower serum Mg^2+^ concentration compared to wildtype littermates. Subsequently, adult *Cnnm2*^+*/−*^ mice were fed with low, control, or high Mg^2+^ diets for two weeks. Adult *Cnnm2*^+*/−*^ mice showed mild hypomagnesaemia compared to *Cnnm2*^+*/*+^ mice and increased serum Ca^2+^ levels, independent of dietary Mg^2+^ intake. Faecal analysis displayed increased Mg^2+^ and Ca^2+^ excretion in the *Cnnm2*^+*/−*^ mice. Transcriptional profiling of *Trpm6*, *Trpm7*, and *Slc41a1* in kidneys and colon did not reveal effects based on genotype. Microcomputed tomography analysis of the femurs demonstrated equal bone morphology and density. In conclusion, CNNM2 is vital for embryonic development and Mg^2+^ homeostasis. Our data suggest a previously undescribed role of CNNM2 in the intestine, which may contribute to the Mg^2+^ deficiency in mice and patients.

## Introduction

Hypomagnesaemia is defined by a serum magnesium (Mg^2+^) level below < 0.7 mmol/L and is associated with hypertension, muscle cramps, diabetes mellitus type II, epilepsy, and cardiac arrhythmias^1^. To maintain a physiological blood Mg^2+^ concentration, the intestine absorbs Mg^2+^ from the diet and can subsequently be stored in soft tissues and bone. The kidneys are considered as major regulators of the blood Mg^2+^ concentration. Here, Mg^2+^ is largely reabsorbed via paracellular transport in the proximal tubule and thick ascending limb of Henle (TAL). Fine-tuning of Mg^2+^ reabsorption take place in the distal convoluted tubule (DCT), where transport is an active transcellular process^2^.

In the DCT, apical Mg^2+^ transport is realised by the luminal divalent cation channel transient receptor potential melastatin type 6 and 7 (TRPM6/7)^3–7^. Mutations in TRPM6 cause hypomagnesaemia with secondary hypocalcaemia (HOMG1/HSH; MIM# 602014). At the basolateral side, several proteins have been considered to facilitate Mg^2+^ extrusion including solute carrier 41a1 (SLC41A1) and Cyclin M2 (CNNM2)^8^. Although the definite role of this latter protein has not been elucidated on a molecular level, patient studies have shown the importance of CNNM2 in renal Mg^2+^ handling^8–11^. Mutations in this gene are causative for hypomagnesaemia, seizures, and intellectual disability (HSMR) syndrome (HSMR; MIM# 616418)^12–14^. In addition to renal Mg^2+^ wasting, patients suffer from seizures, motor skills difficulties, intellectual disability, and deficits in speech^12,13^. Only patients affected by recessive mutations have been demonstrated to show structural brain anomalies, such as demyelination or enlarged supratentorial outer cerebrospinal liquor spaces^13^. Of note, genome wide associates studies (GWAS) have linked the *CNNM2* locus with grey matter morphology, schizophrenia, increased body mass index, and hypertension^15–19^.

Although CNNM2 plays an important role in renal Mg^2+^ homeostasis and brain function, the underlying molecular mechanisms and their implications for patients suffering from HSMR syndrome have not been elucidated. Therefore, the aim of this study was to investigate the role of CNNM2-mediated Mg^2+^ homeostasis in greater detail by generating novel CNNM2 mouse models.

## Methods

### Generation of CNNM2 deficient mice

*Cnnm2*^*−/−*^ mice were generated by CRISPR/Cas9 mediated genomic editing in murine zygotes^20^. In brief, guide RNAs were cloned into pX330 (pX330 was a gift from Feng Zhang (Addgene plasmid 42230) and Cas9 into pTLN^21^. For the generation of Indels in *Cnnm2* the target sequence was 5′-GTCCTGCAAGCAGCTGCGGGC-3′, for insertion of the loxP sites 5′-CTGTAGTAGATCCTCGCGCG-3′ and 5′-GCATCCCGCAGGGTAGATTA-3′. A T7-promoter was added to the sgRNA sequence by PCR and the Cas9 plasmid was linearized using MluI. The PCR product and the linearized plasmid were in vitro transcribed using MEGA shortscript and mMessage Machine KIT (Life Technologies, United States of America (USA)). Both Cas9 mRNA and the sgRNAs were purified using MEGAclear kit (Life Technologies) and eluted in RNase-free water. For the generation of the Indel allele, Cas9 mRNA and sgRNA, for the generation of the floxed allele *Cas9* mRNA, sgRNAs and a plasmid containing a floxed exon 1 of *Cnnm2* were injected into fertilized zygotes. These zygotes were kept overnight (O/N) at 37 °C and then transferred to pseudopregnant foster mice. Founders were identified by PCR and Sanger sequencing. Mice were bred to C57Bl/6N for two generations. The deleted allele was obtained by crossing mice with the floxed allele to a Cre deleter strain (Tg(CMV-cre)1Cgn; JAX #006054).

### Animals and ethics

Two animal studies were performed: a study with embryos and newborn with both *Cnnm2*^+*/*+^, *Cnnm2*^+*/−*^ and *Cnnm2*^*−/−*^ mice and was performed in Charité, Berlin. The other study was performed in the Radboud Institute for Molecular Life Sciences, Nijmegen and used 30 *Cnnm2*^+*/*+^ mice (15 female, 15 male) and 36 *Cnnm2*^+*/−*^ mice (18 female. 18 male). These mice subsequently divided over three experimental groups (*Cnnm2*^+*/*+^ 10 per group, 5 male/female and *Cnnm2*^+*/−*^ 16 mice per group, 6 male/female).

Mice were maintained according to institutional guidelines of Charité Berlin. After timed mating of the mothers, embryos were isolated on day 18.5 of gestation. Newborns were culled directly after birth. Genotyping of the allele containing the deletion was performed using the oligonucleotides 5′-CCGGGTGGGAAGGATGAAGC-3′ and 5′- GGGACCACGTCTCGTTGTTG-3′, and of the floxed allele using the oligonucleotides 5′-AGTCCGGCTCTGGTGCTC-3′ (KO), 5′-AAGCCCAAAACTGCCATTAC-3′ (WT), 5′-TTCTGCCAAAACCACACTTG-3′ (WT/KO). Studies on mice were performed at the Charité Universitätsmedizin Berlin approved by the Animal Care and Use Committee from Berlin:LAGeSo. The study using Mg^2+^ diets in *Cnnm2*^+*/*+^ and *Cnnm2*^+*/−*^ mice was performed at the Radboud Institute for Molecular Life Sciences, Nijmegen, the Netherlands. The study was ethically approved by the Local Ethical Committee of the Radboud University Nijmegen (RU DEC 2015-0112) and by the Dutch Central Commission for Animal Experiments (CCD, AVD103002016382). This study was performed in compliance with the ARRIVE guidelines.

### Experimental design Mg^2+^ diet intervention

Mice were held in standard cages with bedding and kept in a temperature- and light-controlled room. Standard pellet chow and autoclaved tap water were available ad libitum. After genotyping, 36 *Cnnm2*^+*/−*^ mice and 30 *Cnnm2*^+*/*+^ (wild-type) mice of age week 8–10 were evenly divided between females and males, and blood was drawn via submandibular puncture. The mice were then kept in metabolic cages individually for 48 h. Faeces and urine were sampled for 24 h. By genotype and sex, mice were subsequently randomly divided in three diet groups, fed with low 0.02% (w/w) Mg^2+^, normal 0.23% (w/w) Mg^2+^, or enriched 0.48% Mg^2+^ (w/w) diets. After two weeks, mice were housed again in metabolic cages for faeces and urine sampling for 48 h in total and subsequently euthanised by cervical dislocation under 5% (v/v) isofluorance anaesthesia, followed by dissecting the organs.

### Electrolyte measurements

Serum Mg^2+^ and Ca^2+^ concentration of newborn animals were determined by colorimetric assays (QuantiChrom, BioAssay Systems, USA) according to the manufacturer’s instruction).

The total Mg^2+^ concentration in serum, urine, and faeces in adult mice was determined by a xylidyl blue colorimetric assay kit according to the manufacturer's protocol (Roche/Hitachi, Japan) and measured at 600 nm on a BioRad plate reader (BioRad, California, USA). Total Ca^2+^ was measured using an o-cresolphthalein complexone method and read at 570 nm, as described previously^22^. Faeces were dissolved in nitric acid (> 65%) and incubated at 65 °C for 15 min prior to measurements. Sodium and potassium were determined by the Radboudumc clinical lab using an automated analysis system (Abbott Diagnostics, the Netherlands).

### Antibodies

The hexa-His-tagged intracellular carboxyterminus of murine CNNM2 (aa 593-875) was expressed in BL-21 bacteria and purified using a Nickel-nitrilotriacetic acid-column (QIAGEN, Germany). The hexa-His-tag was removed by cleavage with Tobacco Etch Virus protease (Genscript, the Netherlands). Guinea pigs were immunized with the purified protein (Pineda, Germany). Serum was collected after 60 days and 3 boosts and purified by affinity chromatography using the carboxy-terminus of murine CNNM2. Rabbit anti-NCC was a kind gift from Dr. D.H. Ellison. For western blot analyses, rabbit anit-β-actin (A2228, Sigma-Aldrich, USA) and rabbit anti-CNNM2 (ab111351, Abcam, UK) were used.

### SDS-PAGE and Western blot analysis

Tissues were homogenised in 140 mmol/L NaCl, 20 mmol/L Tris, 5 mmol/L EDTA, adjusted with Tris to pH 7.4, with protease inhibitors by Ultra Turrax-T25 followed by Dounce homogeniser. Samples were centrifuged 10 min 1000 g at 4 °C to remove nuclei. Membranes were pelleted by centrifugation at 100,000 g at 4 °C for 30 min and resuspended in lysis buffer (50 mmol/L Tris-HCl pH 6.8, 5 mmol/L EDTA, 2% (w/v) SDS, with protease inhibitors). For SDS-PAGE, 25 µg protein was loaded, followed by transfer to a polyvinylidene fluoride membrane. Membranes were blocked with 5% (w/v) non-fat dry milk in phosphate buffered solution (PBS) for 1 h at room temperature (RT) and incubated overnight with blocking buffer supplemented with primary antibody at 4 °C. The next day, membranes were washed with PBS and incubated with appropriate peroxidase-conjugated secondary antibodies (Roche, Mannheim, Germany) and visualized by enhanced chemiluminescence.

### Histology

Paraffin-embedded brains from adult mice and E17.5 embryos were processed into 5 µm-thick sections and mounted on slides for Nissl staining. Briefly, slides were deparaffinized in xylene for 10 min followed by 5 min incubation in the graded ethanol series (100, 95, 75, 50%). Slides were incubated in 0,1% (w/v) cresyl violet solution prepared in distilled water for 8 mi,n followed by a 2 min differentiation step in 95% ethanol and dehydrated in 100% ethanol for 2 min. Slides were then cleared in xylene and mounted with permanent mounting medium.

### Immunohistochemistry

Kidney cryosections (10 µm) of *Cnnm2*^+/+^ and *Cnnm2*^*−*/*−*^ embryos were fixed with 4% (w/v) paraformaldehyde in PBS for 8 min. Blocking was performed in 5% (v/v) donkey serum (Biozol, Germany), 0.3% (v/v) Triton X-100 in PBS for 1 h at RT. Sections were then incubated in blocking solution supplemented with guinea pig anti-CNNM2 antibody (1:500), and rabbit anti-NCC (1:500) at RT for 2.5 h followed by secondary antibody (AlexaFluor488 donkey-anti-rabbit and AlexaFluor594 donkey-anti-guinea-pig (Thermofisher Scientific, Germany)) incubation for 1 h at RT. Finally, sections were mounted by using ProLong Diamond antifade reagent (Invitrogen, Germany) with DAPI and imaged on Nikon Laser Scanning Microscope A1R (AMBIO core facility, Charité Universtitätsmedizin, Berlin, Germany).

### RNA isolation and real-time quantitative polymerase chain reaction (RT-qPCR)

Total RNA was isolated from kidneys and colon using TriZol (Invitrogen, the Netherlands) and treated with DNAse (1 U/µg RNA, Promega, USA) Subsequently, cDNA was synthesised from 1.5 µg total RNA by Moloney Murine Leukaemia Virus reverse transcriptase (Invitrogen) at 37 °C for 1 h. IQ SYBR Green Mix (Bio-Rad) was used to determine gene expression levels. Relative expression was determined by the Livak method and shown as fold-change compared to the control group (wild-type mice, 0.23% (w/w) Mg^2+^ diet) and corrected for *Gapdh* expression. All primers used for gene expression analysis were equally efficient (see Supplementary Table 1 for primer sequences).

### Structural analysis by the microcomputed tomography

Femurs were dissected and fixed in 4% (v/v) formalin for 24 h and subsequently kept in 70% (v/v) ethanol. Femurs were scanned using the Skyscan 1076 in vivo X-ray computed tomography (Bruker microCT, Kontich, Belgium) with a voxel size of 8.88 μm and 2300 ms exposure time in line with the guidelines for the assessment of bone microstructure^23^. The following settings were used: X-ray power of 40 kV and tube current of 250 mA. Beam hardening (20%) was reduced using a 1 mm aluminum filter, ring-artefacts was set at 5 and an average of three photos (frame averaging) at each angle (0.8°) were taken to generate the final images. For the analysis of trabecular bone parameters, the distal metaphysis was scanned (a scan area of 1.35 mm from the distal growth plate towards femoral center). Analysis of the cortical bone parameters was performed in the diaphyseal cortex, which comprised a scan area of 0.45 mm in the femoral center. 3D reconstruction and data analysis were performed with using manufacturer-provided software (NRecon, Data viewer, CT analyzer; Bruker microCT).

### Statistics

Data is represented as mean ± SEM. Comparisons were made by a One- or- Two-way ANOVA, followed by a Tukey’s post-hoc test. An alpha of *P* < 0.05 was considered statistically significant. Statistical analysis is described in detail in the figure legends.

## Results

### Cnnm2^−/−^ mice are embryonically lethal and display exencephaly

**Figure 1:**
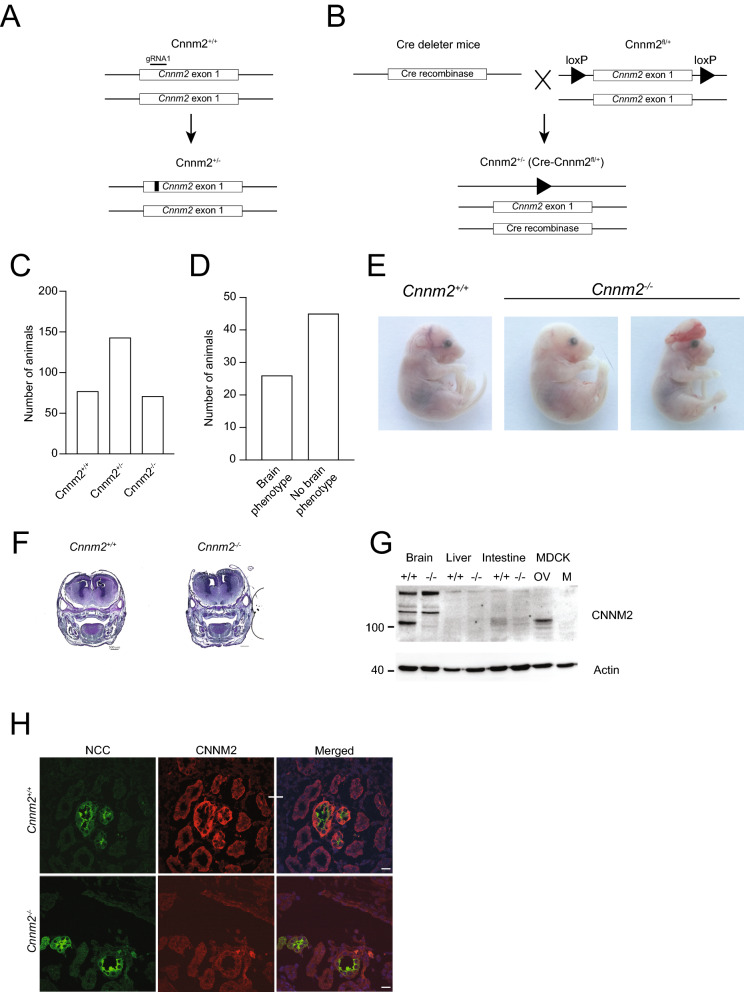
Generation and verification of a *Cnnm2* knock-out mouse model. Schematic overview of the strategy used for genomic deletion of *Cnnm2* by CRISPR/Cas9 mediated deletions (**A**) or by CRISPR/Cas9 mediated insertion of loxP sites followed by Cre recombinase mediated excision (**B**). (**C**) Distribution of the genotype of embryos at E18.5. (**D**) Distribution of *Cnnm2*^*−/−*^ mice exhibiting exencephaly. (**E**) Representative pictures of embryos at E18.5, in which exencephaly is observed in *Cnnm2*^*−/−*^ mice. (**F**) Van Nissl staining of E17.5 *Cnnm2*^+*/*+^ and *Cnnm2*^+*/−*^ embryos showing no apparent differences in morphology in the absence of exencephaly. (**G**) Western blot of brain membrane preparation from *Cnnm2*^+*/*+^ and *Cnnm2*^*−/−*^ mice. Madin-Darby canine kidney cells transfected with murine *Cnnm2* (OV) or mock (M) served as a control for antibody specificity. (**H**) Immunohistochemistry of kidney cryo-sections of E18.5 *Cnnm2*^+*/*+^ and *Cnnm2*^*−/−*^ stained for CNNM2 (red) and Na^+^/Cl^*−*^ co-transporter (NCC) (green). Scale bar depict 50 µm.

*Cnnm2*^*−/−*^ mice were developed using CRISPR/Cas9 technology. In a first approach using Cas9 and a single guideRNA (sgRNA), deletions were introduced in exon 1 of the *Cnnm2* locus. Two independent lines were established with either a deletion of 55 bp resulting in a frameshift at position 28 (p.A28D fsX9) or a deletion of 37 bp resulting in a frameshift at position 40 (p.V40H fsX3) (Fig. 1A). In a second approach using Cas9, two sgRNAs and a donor plasmid, LoxP sites were introduced upstream and downstream of exon 1, which was removed by cre mediated excision after crossing the mice with the floxed allele to a cre deleter strain (Fig. 1B). At E18, embryos were present at Mendelian ratio (*Cnnm2*^+*/*+^: 27%, *Cnnm2*^+*/−*^: 49% *Cnnm2*^*−/−*^: 24%) (Fig. 1C). However, the *Cnnm2*^*−/−*^ offspring all died within the first day after birth in the three investigated lines. Consequently, *Cnnm2*^*−/−*^ animals were analysed at embryonic day 18 (E18). 36% of *Cnnm2*^*−/−*^ embryos displayed exencephaly, which may contribute to the mortality of these mice (Fig. 1D,E). The *Cnnm2*^*−/−*^ embryos (E17.5) that did not show exencephaly also did not show obvious morphological neurological differences, as demonstrated with a Nissl staining (Fig. 1F). Both lines were used to generate mice homozygous for the deleted *Cnnm2* allele and resulted in early postnatal lethality and a similar penetrance of exencephaly.

Absence of CNNM2 protein was confirmed by Western blot analysis of embryonic brains and specificity of the antibody was confirmed using CNNM2 overexpressing Madin-Darby canine kidney (MDCK) cells (Fig. 1G). In addition, immunohistochemistry verified that CNNM2 expression in the DCT, defined by Na^+^/Cl^*−*^ co-transporter (NCC) positive tubules, was absent in kidney sections of *Cnnm2*^*−/−*^ pups (Fig. 1H).

### Cnnm2^+/−^ mice exhibit low serum Mg^2+^ levels

**Figure 2:**
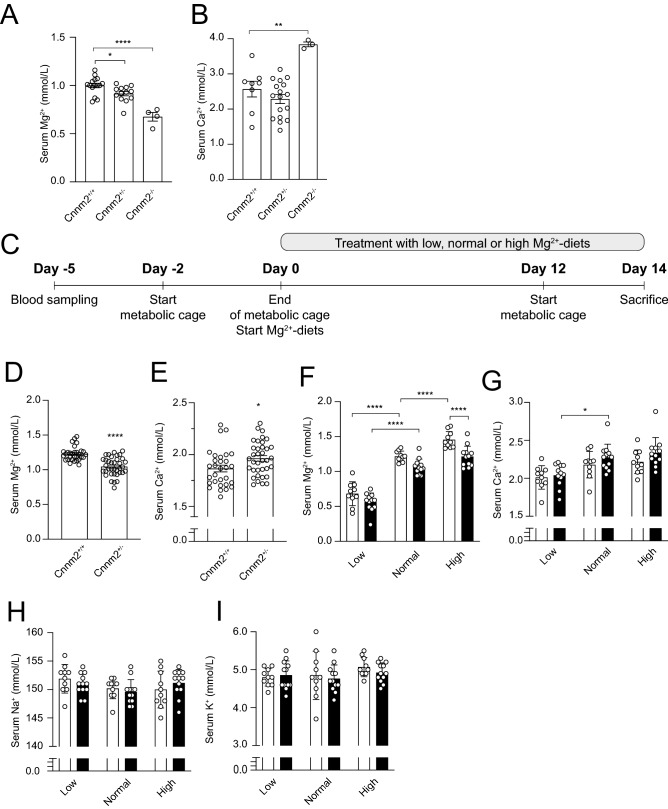
CNNM2 depletion affects Mg^2+^ and Ca^2+^ homeostasis. (**A**) Serum Mg^2+^ is significantly reduced in newborn CNNM2 deficient mice compared to wild type littermates. Data are presented as mean ± SEM (n = 4–15) (**B**) Serum Ca^2+^ in newborn *Cnnm2*^*−/−*^ mice is significantly increased compared to *Cnnm2*^+*/*+^ mice (n = 3–17) (**C**) *Cnnm2*^+*/*+^ or *Cnnm2*^+*/−*^ mice were treated with low (0.02%), normal (0.23%), or high (0.48%) (w/w) Mg^2+^ diets for two weeks, followed by housing in metabolic cages. (**D**,**F**) *Cnnm2*^+*/−*^ mice (black bars) have lower serum Mg^2+^ levels and higher compared to wild type mice (white bars), independent of the Mg^2+^ diet. (**E**,**G**) *Cnnm2*^+*/−*^ mice have lower serum Mg^2+^ levels and an overall increase in serum Ca^2+^ levels compared to wild type littermates, independent of the Mg^2+^ diet. Serum Na^+^ (**H**) and K^+^ (**I**) levels in *Cnnm2*^+*/−*^ mice are unchanged (n = 10–12 per group). Data are presented as mean ± SEM. Significance determined with two-tailed Student’s T-test or Two-way ANOVA followed by a Tukey post-hoc test. *p < 0.05, **p < 0.01, ***p < 0.001, ****p < 0.0001.

To determine the effects of CNNM2 depletion on electrolyte homeostasis, serum Mg^2+^ and Ca^2+^ levels were determined in pups within the first hours of life (≤ 8 h). The *Cnnm2*^*−/−*^ mice had a significant decreased serum Mg^2+^ concentration compared to both *Cnnm2*^+*/−*^ mice and *Cnnm2*^+*/*+^ mice (0.68 ± 0.05 mmol/L versus 0.92 ± 0.02 mmol/L and 1.00 ± 0.02 mmol/L, respectively, P < 0.05) (Fig. 2A). Determination of the serum Ca^2+^ levels revealed a significant 50% increase in *Cnnm2*^*−/−*^ mice whereas similar Ca^2+^ levels were observed in *Cnnm2*^+*/−*^ mice (Fig. 2B).

As *Cnnm2*^*−/−*^ mice died shortly after birth, further studies to determine the role of CNNM2 in Mg^2+^ homeostasis were performed in the adult *Cnnm2*^+*/−*^ mice. Adult *Cnnm2*^+*/−*^ had comparable brain morphology with wild type mice (Supplementary Fig. 2).

To delineate the role of CNNM2 in Mg^2+^ further, *Cnnm2*^+*/*+^ and *Cnnm2*^+*/−*^ adult mice were fed with a low, normal, or high Mg^2+^ diet for two weeks (Fig. 2C). Similar to newborn *Cnnm2*^*−/−*^ mice*,* adult *Cnnm2*^+*/−*^ mice displayed significantly decreased Mg^2+^ levels in the blood compared to *Cnnm2*^+*/*+^ mice (1.05 ± 0.02 versus 1.23 ± 0.02 mmol/L, respectively, *P* < 0.05) (Fig. 2D). When exposed to a low, normal, or high Mg^2+^ diet for two weeks, serum Mg^2+^ levels were consistently low post-diet in *Cnnm2*^+*/−*^ mice compared to *Cnnm2*^+*/*+^ (Fig. 2E). No differences were observed in weight gain between mice of different genotypes or when fed on a particular diet (Table 1). In adult *Cnnm2*^+*/−*^ mice, a slight increase of serum Ca^2+^ concentration was measured compared to *Cnnm2*^+*/*+^ mice (Fig. 2F,G). Blood Na^+^ and K^+^ levels remained unaffected upon exposure to the diets (Fig. 2H,I).

**Table 1:** Metabolic parameters of Cnnm2^+/*−*^ mice upon exposure to different Mg^2+^ diets.

Diet	*Cnnm2*^+*/*+^			*Cnnm2*^+*/−*^		
	Low	Normal	High	Low	Normal	High
Body weight M (g)	23.7 ± 0.3	22.8 ± 0.3	24.4 ± 0.3	23.3 ± 0.4	23.7 ± 0.8	23.7 ± 0.5
Body weight F (g)	19.5 ± 0.8	19.5 ± 0.5	19.7 ± 0.4	20.1 ± 0.4	20.3 ± 0.2	20.7 ± 0.3
Food intake (g)	3.5 ± 0.1	3.0 ± 0.2	3.4 ± 0.2	3.5 ± 0.2	3.5 ± 0.2	3.2 ± 0.3
Water intake (mL)	10 ± 2.4	9.7 ± 1.8	10 ± 1.7	10 ± 1.6	10 ± 1.3	7.9 ± 0.9
Urine excretion (mL/24 h)	3.4 ± 0.9	2.3 ± 0.4	3.8 ± 0.7	3.7 ± 0.7	3.3 ± 0.4	3.5 ± 0.6
Faecal (mg/24 h)	384 ± 21	429 ± 40	450 ± 36	391 ± 40	442 ± 38	428 ± 35

*Cnnm2*^+*/*+^ and *Cnnm2*^+*/−*^ mice were kept in metabolic cages exposed at low (0.02%), normal (0.21%) and high (0.48%) (w/w) Mg^2+^ diets for 14 days. The last 48 h, mice were kept in metabolic cages for the collection of urine and faeces. Values represent the latter 24 h (mean ± SEM). *M* male, *F* female.

### Cnnm2 deficiency leads to mild urinary Mg^2+^ wasting

**Figure 3:**
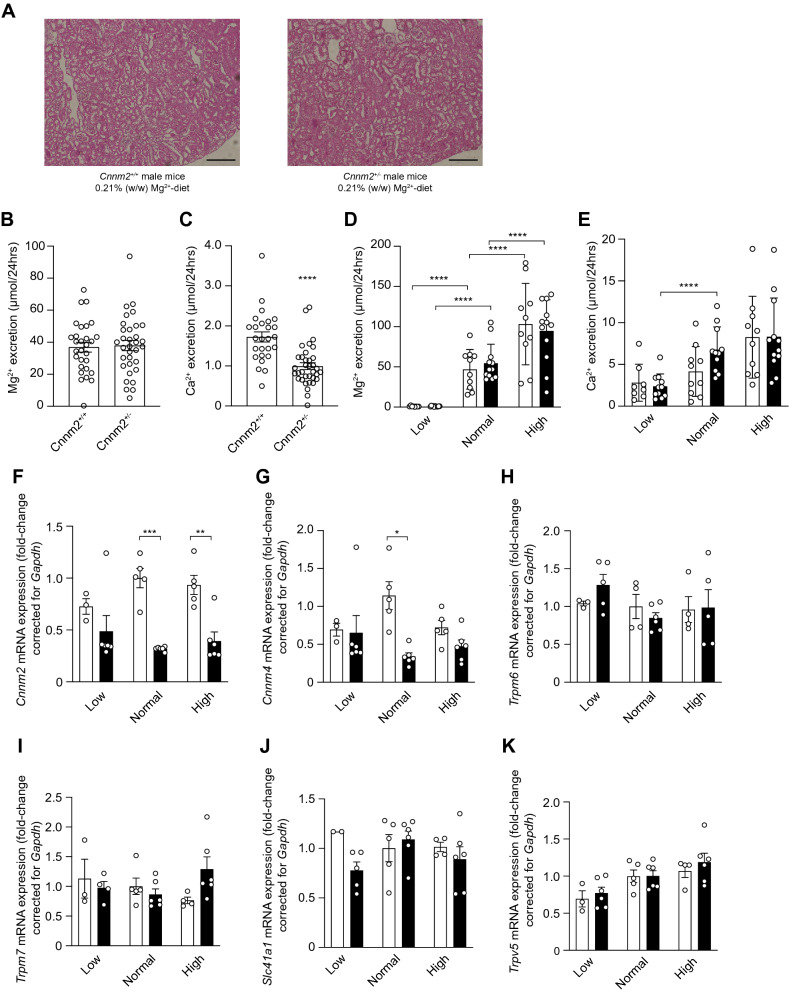
*Cnnm2* deficiency does not affect overall renal Mg^2+^ reabsorption. (**A**) Representative pictures of kidney morphology by H&E staining. Scale bar depicts 50 μm. (**B**,**D**) *Cnnm2*^+*/−*^ mice do not show altered Mg^2+^ excretion via the urine, but retain more Ca^2+^ (n = 30–36 per group). (**C**–**E**) Lower Mg^2+^ intake is associated with lower renal Mg^2+^ excretion, not with Ca^2+^, independent of genotype in *Cnnm2*^+*/−*^ mice (black bars) (n = 8–12 per group). (**F**) Renal *Cnnm2* expression is lowered in *Cnnm2*^+*/−*^ mice, *Cnnm4* (**G**), *Trpm6* (**H**), *Trpm7* (**I**), *Slc41a1* (**J**), and *Trpv5* (**K**) remain majorly unaltered (n = 3–6 mice per group). Data are presented as mean ± SEM. Significance determined with two-tailed Student’s T-test or Two-way ANOVA followed by a Tukey post-hoc test. *p < 0.05, **p < 0.01, ***p < 0.001, ****p < 0.0001.

To pinpoint the cause of the low serum Mg2 + levels in the *Cnnm2*^+*/−*^ mice, the 24 h urinary Mg^2+^ excretion and kidney mRNA expression of genes involved in Mg^2+^ reabsorption were assessed. Morphology of the kidneys was similar in both genotypes (Fig. 3A). *Cnnm2*^+*/−*^ deficient mice did not show a significant decrease in urinary Mg^2+^ excretion (38 ± 3.2 versus 37 ± 3.0 mmol/L/24hrs, respectively) in the urine compared to wild type mice across the different diets (Fig. 3B,C), despite having decreased serum Mg^2+^ concentration, suggestive for a small renal reabsorption defect. Interestingly, *Cnnm2*^+*/−*^ displayed Ca^2+^ retention at the baseline urine measurement (1.0 ± 0.1 vs 1.7 ± 0.1 μmol/24hrs, respectively, *P* < 0.0001) (Fig. 3D), although this effect was not observed at the final day of the diets (Fig. 3E). Gene expression analysis showed significantly decreased renal expression of *Cnnm2* in *Cnnm2*^+*/−*^ mice on the control and high Mg^2+^ diet (Fig. 3F). Expression of the family member *Cnnm4* was decreased 3.6-fold in *Cnnm2*^+*/−*^ mice fed on the normal Mg^2+^-diet (Fig. 3G). Levels of magnesiotropic genes *Trpm6*, *Trpm7*, *Slc41a1* were measured in the kidney, but did not show any genotype-specific effect (Fig. 3H–J). The expression of the epithelial Ca^2+^ channel *Trpv5* showed an overall significant increase due the diets, but no differences were observed between the *Cnnm2*^+*/−*^ compared to wild type mice (Fig. 3K).

### Cnnm2^+/−^ mice have perturbed intestinal Mg^2+^ absorption

**Figure 4:**
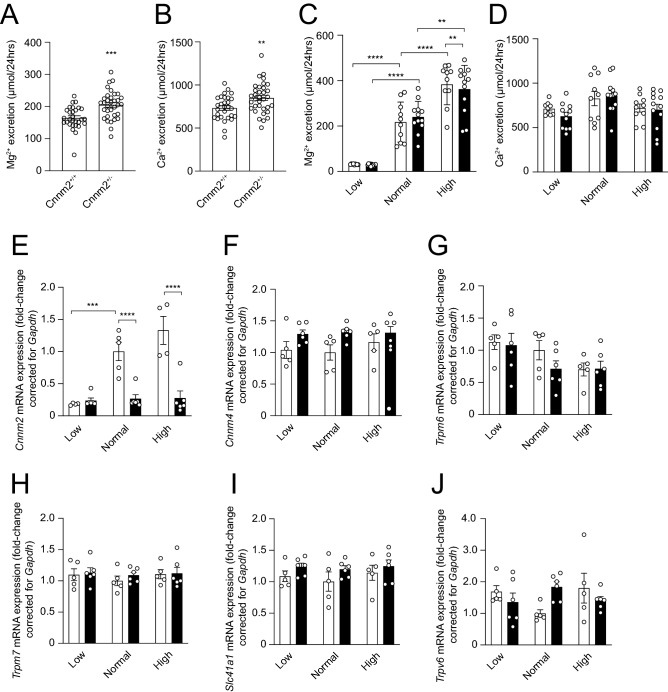
*Cnnm2* deficiency decreases faecal Mg^2+^ absorption. (**A**,**C**) *Cnnm2*^+*/−*^ mice display significantly increased Mg^2+^ and Ca^2+^ excretion via the faeces (n = 29–34 per group). (**B**,**D**) Lower Mg^2+^ intake is associated with lower intestinal Mg^2+^ excretion, not with Ca^2+^, independent of genotype in *Cnnm2*^+*/*+^ (white bars) and *Cnnm2*^+*/−*^ mice (black bars) (n = 8–12 per group) (**E**) *Cnnm2* expression is significantly diminished in *Cnnm2*^+*/−*^ mice, *Cnnm4* (**G**), *Trpm6* (**H**), *Trpm7* (**I**), *Slc41a1* (**J**), and *Trpv6* (**K**) remain unaltered (n = 3–6 per group). Data are presented as mean ± SEM. Significance determined with two-tailed Student’s T-test or Two-way ANOVA followed by a Tukey post-hoc test. *p < 0.05, **p < 0.01, ***p < 0.001, ****p < 0.0001.

Before the start of the diets *Cnnm2*^+*/−*^ mice displayed a 17.5% higher faecal Mg^2+^ content compared to *Cnnm2*^+*/*+^ mice (Fig. 4A,B). This was similar to Ca^2+^ levels, which were also significantly increased (Fig. 4C,D). Furthermore, we investigated the mRNA expression of key magnesiotropic genes present in the distal part of the colon, where transcellular Mg^2+^ absorption takes place. Similar to the observation in the kidney, *Cnnm2* transcript levels were significantly reduced in the *Cnnm2*^+*/−*^ mice compared to control mice. Yet there was an overall increase of *Trpm6* expression and *Cnnm4* expression based on diet or genotype, respectively (Two-Way ANOVA). No diet or genotype dependent changes were observed for other magnesiotropic genes (Fig. 4E–I). The expression of the epithelial Ca^2+^ channel *Trpv6* was not significantly different among all mice groups (Fig. 4J).

### Cnnm2^+/−^ mice display normal bone morphology

**Figure 5:**
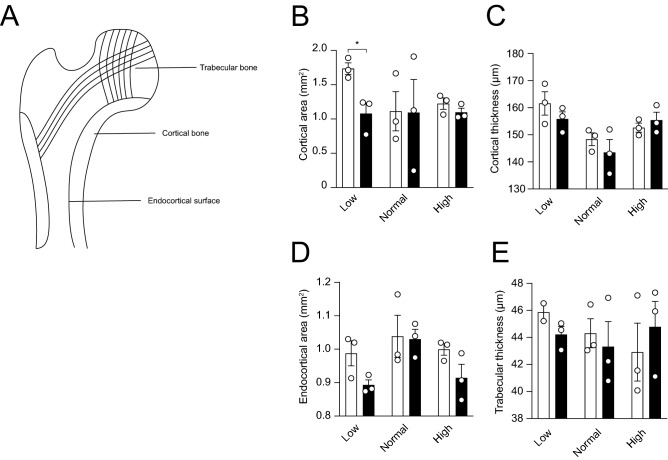
CNNM2 depletion does not affect bone morphology. Micro-CT analysis of femurs of which the cortical area (**A**) and thickness (**B**) were determined, endocortical area (**C**) and trabecular thickness (**D**). (n = 3 per group) Data are presented as mean ± SEM. Significance determined with two-tailed Student’s T-test or Two-way ANOVA followed by a Tukey post-hoc test. *p < 0.05.

The femurs of three male mice were subjected to micro-computed tomography analysis. Both the cortical and trabecular bone microarchitecture were not significantly affected by genotype nor diet, except in *Cnnm2*^+*/−*^ mice on a low Mg^2+^ diet (Fig. 5A–E).

## Discussion

In this study, we used several genetic mouse models to investigate the role of CNNM2 in mice. *Cnnm2*^*−/−*^ mice exhibited maldevelopment of the brain, prominently consisting of exencephaly. The *Cnnm2*^*−/−*^ newborns displayed hypomagnesaemia concomitant with increased serum Ca^2+^ levels. *Cnnm2*^+*/−*^ adult mice showed a normal development, but similarly suffered from a low serum Mg^2+^ concentration, which could be partially explained by faecal and renal Mg^2+^ loss. Our study emphasises the role of CNNM2 in embryonic development and overall Mg^2+^ handling, and disclosed a novel role in intestinal Mg^2+^ absorption.

Impaired renal Mg^2+^ reabsorption and urinary Mg^2+^ wasting have been considered as the primary defect of HSMR patients as renal Mg^2+^ excretion remains similar despite lowered serum Mg^2+^ levels^13,14^. In line with the observation in patients, *Cnnm2*^+*/−*^ mice displayed low serum Mg^2+^ levels when compared to *Cnnm2*^+*/*+^ mice. Yet, no significant increase in urinary Mg^2+^ excretion was observed. The *Cnnm2*^+*/−*^ mice displayed lowered urinary Mg^2+^ excretion upon exposure to lowered Mg^2+^ diet, similarly to wild type mice. This demonstrates the compensatory capacity of Mg^2+^ reabsorption in these mice. Yet, *Cnnm2*^+*/−*^ mice showed decreased serum Mg^2+^ levels. The fact that this low serum value is not associated with a diminished urinary Mg^2+^ excretion can be interpreted as a sign of a renal Mg^2+^ leak. This has previously been reported in other mouse models of hypomagnesemia, e.g. *Slc41a3*^*−/−*^ mice and high-fat diet-fed mice^24,25^. The role of CNNM2 in the kidney has been substantiated by studies of kidney-specific knockout mice of *Cnnm2* which suffer from hypomagnesemia, demonstrating that CNNM2 regulates renal Mg^2+^ handling^19^.

Interestingly, intestinal malabsorption may also play a role in the Mg^2+^ deficiency observed in the *Cnnm2*^+*/−*^ mice. At basal conditions, faecal Mg^2+^ excretion was increased in *Cnnm2*^+*/−*^ mice, indicative of Mg^2+^ malabsorption. Although CNNM2 has been primarily studied in the kidney, our current and previous studies demonstrate that *Cnnm2* is expressed in the distal colon^8^. Indeed, several recent single cell RNA sequencing datasets in colon and rectum confirm that CNNM2 is particularly expressed in enterocytes^26,27^. Of interest, CNNM4, a close family member of CNNM2, is present in the basolateral membrane of the colon, where it orchestrates Mg^2+^ efflux^28–30^. We have previously shown an interaction of CNNM2 with CNNM4 upon expression in HEK293 cells, suggesting that these proteins could form a complex^8^. Indeed, a significant increase of *Cnnm4* expression in the colon was observed in *Cnnm2*^+*/−*^ mice, suggesting that CNNM4 compensates for the loss of CNNM2. However, a CNNM4-independent function of CNNM2 cannot be excluded. Although malabsorption of Mg^2+^ was not previously reported in *Cnnm2*^+*/−*^ mice or patients^19^, it should be noted that faecal Mg^2+^ excretion is often not detected or even determined in mice, although also in intestinal-specific *Trmp6*^*−/−*^ and *Trpm7*^*−/−*^ mice decreased serum Mg^2+^ levels and increased faecal Mg^2+^ excretion can be observed^6,31^. On average, 30% of dietary Mg^2+^ is absorbed and foremost ends in the faeces, making it challenging to detect changes in absorption. However, Mg^2+^ supplementation was proven difficult in patients with *CNNM2* mutations, which potentially indicates a reduced ability to absorb Mg^2+^^13,14^. Our findings suggest that oral Mg^2+^ supplementation in patient with HSMR syndrome depends on paracellular Mg^2+^ transport in the duodenum, as transcellular Mg^2+^ absorption in the colon may be reduced by CNNM2 mutations.

In addition to the Mg^2+^ disturbances, *Cnnm2*^+*/−*^ mice displayed renal Ca^2+^ retention resulting in an increased serum Ca^2+^ concentration. Decreased serum Mg^2+^ levels could trigger the TAL to increase its paracellular Mg^2+^ transport. In addition, this would lead to increased Ca^2+^ reabsorption and decreased urinary Ca^2+^ levels, which is coordinated via Claudins 10, 14, 16 and 19^32–36^. This compensatory capacity may be specific for mice, as patients with mutated *CNNM2* have significant urinary Mg^2+^ wasting. In line with this, 24 h urinary Ca^2+^ levels are normal in patients with *CNNM2* mutations. Interestingly, a few patients with *CNNM2* mutations have been reported with impaired Ca^2+^ and phosphate homeostasis. Whether this is a common phenotype in these patients remains to be determined, as the patients are consanguineous or have other genetic defects, such as mutations in the vitamin D receptor (VDR)^12,37^. However, the role of TAL in CNNM2-associated physiology should be interpreted with caution, as direct effects of CNNM2 on Ca^2+^ homeostasis cannot be excluded.

Our study demonstrates that CNNM2 is essential for brain development and early life survival. Until embryonic day 18.5, the *Cnnm2*^*−/−*^ embryos were present at Mendelian ratios, but newborn *Cnnm2*^*−/−*^ mice died shortly after birth. Although lethality of *Cnnm2*^*−/−*^ mice was reported earlier^19^, we are the first to report the presence of exencephaly, which suggests that neural tube defects contribute to the brain phenotype. It is known that defects in the closure of the cranial neural tube result in protrusion of the neuroepithelium outside the developing brain, inevitably disturbing normal neurodevelopment^38^. Although previous studies suggested an important role of CNNM2 in brain function, the exact consequences of CNNM2 deficiency may be species-dependent. Patients with recessive CNNM2 mutations exhibit structural brain deformities, such as demyelination, failure of opercularisation, and cerebral cortical atrophy, often concomitant with motor skill defects, epileptic seizures, and intellectual disability^12,13^. Moreover, knockdown of cnnm2 in zebrafish resulted in maldevelopment of the mid-hindbrain boundary^13^.

Interestingly, exencephaly and other neural tube defects have been more often observed in mouse models of hypomagnesemia. *Trmp6* knockout mice display embryonic lethality, which is accompanied by the presence of exencephaly and spina bifida^5,39^. Similarly, the absence of *Trpm7* expression in mice during embryonic development, a close homologue of TRPM6, has been associated with neural tube defects^40,41^. Although this homology suggests that neural tube defects may be the direct result of hypomagnesaemia, it should be noted that neural tube defects are uncommon in patients with hypomagnesaemia^1^. Consequently, it remains to be determined whether this is a Mg^2+^ dependent or independent effect. Of note: as only a subset of *Cnnm2*^*−/−*^ mice displayed exencephaly, neural tube defects may only partially explain the lethality.

The absence of a clear urinary Mg^2+^ wasting in *Cnnm2*^+*/−*^ mice shows that renal Mg^2+^ reabsorption may be different in mice and men, as HSMR syndrome show clear renal Mg^2+^ wasting^13^. In line with this, kidney-specific *Trpm6* knock out mice were reported to have normal serum Mg^2+^ levels and without renal Mg^2+^ leak, unlike patients with *Trpm6* mutations (HOMG1/HSH; MIM# 602014)^31^. Similarly, *Kcnj10* and *Fxyd2* knock out animals have normal urinary Mg^2+^ levels, in contrast to patients with mutations (OMIM: 612780 & 154020)^42–46^. Additionally, a mice model deficient in solute carrier family 41 member 3 (*Slc41a3*) expression, a putative Mg^2+^ transporter and highly enriched in DCT, was associated with hypomagnesemia without renal Mg^2+^ wasting^24^. Altogether, these studies show that mice are able to compensate better for impaired DCT Mg^2+^ reabsorption compared to men.

In conclusion, CNNM2 regulates the systemic Mg^2+^ balance and is essential for neurodevelopment. Our study points toward a putative role of CNNM2 in intestinal Mg^2+^ absorption.

## Supplementary Information

Supplementary Information.
