# Supplementary material for: Cyclin M2 (CNNM2) knockout mice show mild hypomagnesaemia and developmental defects
Source: Sci Rep. 2021 Apr 15;11:8217. doi: 10.1038/s41598-021-87548-6 (PMC8050252; doi:10.1038/s41598-021-87548-6)
Supplement: Supplementary file 1 — Supplementary Information. [file 41598_2021_87548_MOESM1_ESM.pdf]

Cyclin M2 (CNNM2) knockout mice show mild hypomagnesaemia and developmental defects

Franken, Gijs A.C.[1*], Seker, Murat[2*], Bos, Caro[1], Siemons, Laura A.H. [1], van der Eerden, Bram C.J.[3], Christ, Annabel[4] Hoenderop, Joost G.J.,[1], Bindels, René J.M.[1], Müller, Dominik[2], Breiderhoff, Tilman[2], de Baaij, Jeroen H.F[1].

* These authors contributed equally to this work

[1] Department of Physiology, Radboud Institute for Molecular Life Sciences, Radboud university medical center, Nijmegen, the Netherlands.

[2] Department of Pediatric Gastroenterology, Nephrology and Metabolic Diseases, Charité – Universitätsmedizin Berlin, Berlin, Germany

[3] Department of Internal Medicine, Erasmus Medical Center, Rotterdam, the Netherlands

[4] Department of Molecular Cardiovascular Research, Max-Delbrueck-Center for Molecular Medicine, Berlin, Germany.

Supplementary table 1: Primer sequences for real-time quantitative PCR

Gene	**Forward primer (5'-3')**	**Reverse primer (5'-3')**
Gapdh	AAGTGGAGATTGTTGCCATC	GTTGTCATGGATGACCTTGG
Cnnm2	GTCTCGCACCTTTGTTGTCA	GTCGCTCCGACTGAGAGAAT
Cnnm4	TCTGGGCCAGTATGTCTCTG	CACAGCCATCGAAGGTAGG
Trpm6	AAAGCCATGCGAGTTATCAGC	CTTCACAATGAAAACCTGCCC
Trpm7	GGTTCCTCCTGTGGTGCCTT	CCCCATGTCGTCTCTGTCGT
Slc41a1	TCCCTGATGGCCACTTTAGC	GATCATACCCAGGACCAAGGAG
Trpv5	CTGGAGCTTGTGGTTTCCTC	TCCACTTCAGGCTCACCAG
Trpv6	GGCCTCACAACCTCATTTAC	CTCAATGAGCAGTCTAACAATC

Supplementary Figure 1

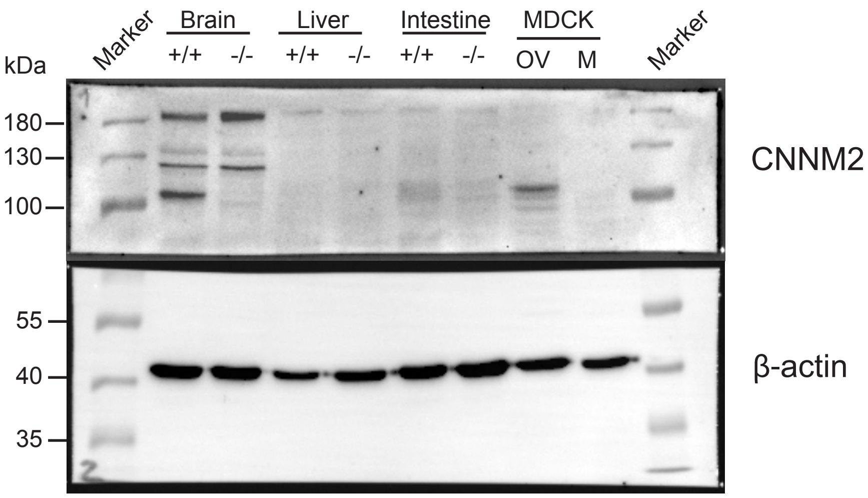

Supplementary Figure 2

Cnnm2$^{+/+}$

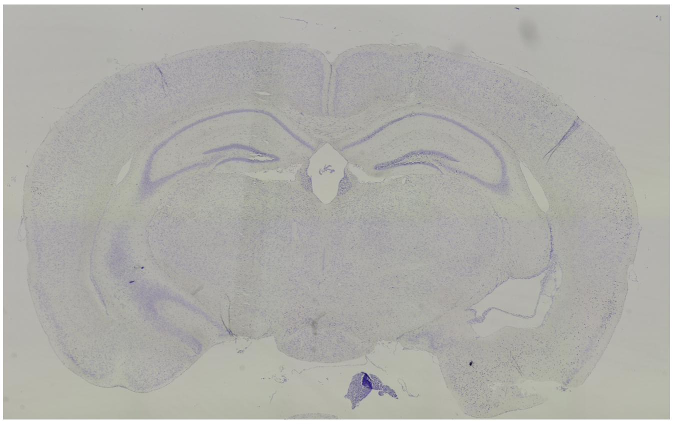

Cnnm2$^{+/-}$

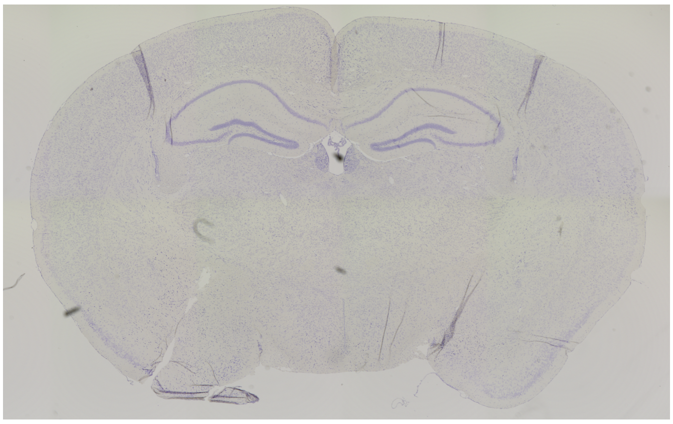

Supplementary Figure 1

Full Western Blot (overlay of chemiluminescence and epi-white) of brain, liver, and intestine membrane preparations from *Cnnm2*$^{+/+}$ and *Cnnm2*$^{-/-}$ mice (Figure 1G). MDCK cells transfected with murine *Cnnm2* (OV) or mock (M) served as a control for antibody specificity. B-actin was used as control.

Supplementary Figure 2

Nissl staining of the brain of *Cnnm2*$^{+/+}$ and *Cnnm2*$^{+/-}$ adult mice showing normal, comparable morphology.
